# 

**Published:** 1869-08-15

**Authors:** 


### CHICAGO MEDICAL JOURNAL.

J ADAMS ALLEN, M.D., LL.D., WALTER HAY, A.M., M.D., Editors.

All business letters for THE JOURNAL should be addressed to J. WOLCOTT HOOKER, Room 12, Union Building, Chicago, Ill.

All communications for publication should be addressed to DR. J. ADAMS ALLEN, 503 Michigan Avenue, Chicago, Ill.

TERMS—$3 PER ANNUM, IN ADVANCE.

$4 if not paid before 1st of July, in each year. Single Copies, 25 Cents.

### CHICAGO

### WHOLESALE PRICE-CURRENT

*Of Drugs, Medicines, Chemicals, Etc.*

CORRECTED BY

E. P. DWYER & Co.

| | | |
|---|---|---|
| Acid, Acetic. by carboy ........ | per lb. | $0 80 |
| Less than carboy ........... | " | 25 |
| Benzoic ......................... | per oz. | 40 |
| Carbolic, cryst.................. | per lb. | 1 75 |
| do solution........... | " | 60 |
| Citric, by keg, 112 lbs...... | " | 1 12 |
| Less than keg .............. | " | 1 20 |
| Fluoric........................ | " | 3 00 |
| Gallic, bottle included...... | per oz. | 35 |
| Muriatic, by carboy ......... | per lb | 6 |
| Less than carboy............ | " | 8 |
| Nitric, 41 per cent., by carboy.......................... | " | 18 |
| Less than carboy............ | " | 20 |
| Nitric 38 per cent. by carboy ........... ............ | " | 16 |
| Less than carboy............ | " | 20 |
| Oxalic .......................... | " | 36 |
| Sulphuric, by carboy........ | " | 4¼ |
| Less than carboy............ | " | 7 |
| Tartaric, powdered, P. & W. by 50 lb. case ............. | " | 80 |
| Less than case ............... | " | 85 |
| Aloes, Cape ........................ | " | 25 |
| Cape, powdered .............. | " | 36 |
| Barbadoes ...................... | " | 60 |
| Socotrine........................ | " | 1 05 |
| Socotrine, powdered......... | " | 1 20 |
| Ammonia, Carbonate............ | " | 25 |
| Muriate, by bbl............... | " | 17 |
| Less than bbl ................. | " | 20 |
| Aqua, 4 F........................ | " | 12 |
| do do by carboy......... | " | 10 |
| do 3 F........................ | " | 10 |
| do do by carboy......... | " | 8 |
| Bromide........................ | " | 2 75 |
| do .......................... | per oz. | 25 |
| Antimony, powdered, black, pure, by 100 lb case...... | per lb. | 6½ |
| Less than case................ | " | 8 |
| Aniseed, Italian.................. | " | 26 |
| Star ............................. | " | 55 |
| Arnica Flowers .................... | " | 28 |
| Arrow Root, American.......... | " | 15 |
| Barmuda ........................ | " | 55 |
| St. Vincent..................... | " | 25 |
| Taylor's in foil, by 12 lb. case.......................... | " | 50 |
| Less than case................ | " | 55 |
| Arsenic, by keg.................... | " | 6 |
| Less than keg ................ | " | 10 |
| Balsam Copaiva, pure............ | " | 1 10 |
| Fir ............................... | " | 55 |
| Fir ................................. | per gal. | 4 00 |

| | | |
|---|---|---|
| Balsam Peru ......................... | per lb. | 5 50 |
| Tolu ............................. | " | 1 40 |
| Sulphur ........................ | " | 50 |
| Barbadoes, Tar True.............. | " | 20 |
| Barley, Pearl, by keg............ | " | 9 |
| Less than keg.................. | " | 10 |
| Bayberry Bark...................... | " | 15 |
| Powdered...................... | " | 20 |
| Bay Rum, pure, imported........ | per gal. | 6 25 |
| Commercial.................... | " | 4 00 |
| Bismuth, Subnitrate, P. & W... | per lb. | 7 50 |
| Bisulphuret Carbon................ | " | 45 |
| Blue Pill, P. &W,.................. | " | 85 |
| English........................ | " | 1 00 |
| Blue Vitriol, by bbl.............. | " | 15 |
| Less than bbl.................. | " | 16 |
| Borax, refined, by bbl........... | " | 33 |
| Less than bbl.................. | " | 36 |
| Powdered...................... | " | 45 |
| Brimstone, by bbl................. | " | 4¾ |
| Less than bbl ................. | " | 5½ |
| Bromine, vial included ........... | per oz. | 55 |
| Buchu Leaves, long ............... | per lb. | 60 |
| Short............................ | " | 40 |
| Burgundy Pitch, True........... | " | 16 |
| American...................... | " | 12 |
| Calabar, Beans....................... | per oz. | 75 |
| Calamus Root........................ | per lb. | 25 |
| Bleached, No. 1 ............. | " | 60 |
| do No. 2............ | " | 40 |
| Calomel, P. & W., bulk........... | " | 1 05 |
| P. & W., bottles.............. | " | 1 10 |
| English (genuine)............ | " | 1 50 |
| Camphor Gum, refined ........... | " | 95 |
| Canary Seed, by bag............... | " | 10 |
| Less than bag.................. | " | 11 |
| Cantharides.......................... | " | 2 00 |
| Powdered....................... | " | 2 15 |
| Capsicum, African, powdered.. | " | 42 |
| Caraway Seed........................ | " | 24 |
| Cardamon Seed, Madras......... | " | 4 00 |
| Malabar ......................... | " | 4 50 |
| Cassia, in Mats...................... | " | 80 |
| Buds ............................ | " | 1 15 |
| Caustic Soda, best brands........ | " | 8¼ |
| Celery Seed .......................... | " | 70 |
| Cerate Cantharides................ | " | 1 15 |
| Chalk, White, by bbl.............. | " | 2¾ |
| Less than bbl.................. | " | 4 |
| Crayons ........................ | per box | 35 |
| Red Fingers.................... | per lb. | 8 |
| Prep. Drops ................... | " | 9 |
| Precip. English ............... | " | 25 |

| | | |
|---|---|---|
| Chamomile Flowers, Roman, new | per lb. | 50 |
| Chamomile Flowers, German | " | 40 |
| Chinoidine, P. & W., oz. rolls | per oz. | 20 |
| Chloride of Lime, bycask | per lb. | 5 |
| Less than cask | " | 7 |
| Chloroform, P. & W. | " | 1 45 |
| Squibb's | " | 3 00 |
| Citrine Ointment, P. & W. | " | 50 |
| Civette | per oz. | 8 00 |
| Cloves | per lb. | 44 |
| Powdered | " | 60 |
| Cobalt, powdered | " | 35 |
| Cochineal, Hond, genuine | " | 1 35 |
| L. S. | " | 1 25 |
| Cocoa, butter | " | 1 00 |
| Colchicum Root, English | " | 25 |
| Seed | " | 25 |
| Collodion | " | 1 85 |
| Colocynth Apple | " | 70 |
| Powdered | " | 75 |
| Columbo Root, American | " | 25 |
| Foreign | " | 24 |
| Powdered | " | 35 |
| Composition Powder, 2 oz. packages | " | 45 |
| Concentrated Lye (old style iron case) | per case | 8 25 |
| New style paper box | " | 6 50 |
| Confection Rose | por lb. | 55 |
| Senna | " | 55 |
| Copperas, by bbl | " | 2¼ |
| Less than bbl | " | 3½ |
| Coriander Seed | " | 16 |
| Corrosive Sublimate | " | 95 |
| Crab Orchard Salts | " | 40 |
| Cream Tartar, perfectly pure, powdered, Grocer's | " | 30 @ 40 |
| Cubebs, berries | " | 38 |
| Powdered | " | 40 |
| Cudbear No. 1 | " | 42 |
| No. 2 | " | 25 |
| Cumin Seed | " | 18 |
| Cuttle Fish Bone | " | 35 |
| Dragon's Blood, in Mass | " | 1 00 |
| Drug Mills, Swift's, *Ea* | — | 15 00 |
| Dover's Powders, P. & W. | " | 3 20 |
| Elm Bark, select | " | 15 |
| Powdered | " | 20 |
| Ground | " | 18 |
| Emery Grains, all Nos | " | 12 |
| Flour, by keg | " | 8 |
| Less than keg | " | 10 |
| Epsom Salts, by bbl | " | 4¾ |
| Less than cbl | " | 5½ |
| Ergot | " | 1 35 |
| Powdered | " | 1 50 |
| Ether Sulphuric | " | 70 |
| Washed | " | 75 |
| Concentrated | " | 85 |
| Acetic | " | 90 |
| Chloric | " | 1 00 |
| Extract, Belladonna, English | " | 4 05 |
| Cannabis Indica, English | per oz. | 1 50 |
| Conium, English | per lb. | 2 00 |
| Dandelion, English | " | 1 90 |
| Hyosciamus, English | " | 5 00 |
| Fennel Seed | " | 18 |
| Flax Seed | " | 7 |
| Ground, by bbl | " | 7 |
| Less than bbl | " | 7½ |
| Fœnugreek Seed, powdered | " | 16 |
| Gambier, or Eerra Japonica, by bale | " | 7½ |
| Less than bale | " | 10 |
| Gelatine, Coxe's | per doz. | 2 50 |
| Pink, French | per lb. | 1 60 |

| | | |
|---|---|---|
| White | per lb. | 65 |
| Gelatine, Cooper's, by 12 lb. boxes | " | 90 |
| Less thon 12 lb. box | " | 95 |
| Gentian Root | " | 20 |
| Powdered | " | 25 |
| Ginger Root, Jamaica, bleached | " | 35 |
| Jamaica, bloached, powdered | " | 40 |
| E. I., powdered, pure | " | 26 |
| E. I., Root | " | 22 |
| Glauber Salts, by bbl | " | 2½ |
| Less than bbl | " | 3½ |
| Glue, A., Extract, Cooper's, White | " | 55 |
| B. & A., White | " | 50 |
| No. 1 Extra Cooper's, White | " | 48 |
| No. 1 Sheet | " | 25 |
| No. 2 Sheet | " | 22 |
| Common | " | 18 @ 21 |
| Glycerine, Bowers' | " | 80 |
| Heavy, Inodorous. | " | 45 |
| Price's English | " | 1 10 |
| P. & W., No. 1, concentrated | " | 45 |
| P. & W.. No. 2, concentrated | " | 35 |
| Proctor & Gamble's | " | 50 |
| Vienna, 1 lb. bottles | " | 45 |
| Gum Aracic, 1st select | " | 95 |
| 2d select | " | 70 |
| 3d select | " | 60 |
| Prime, sorts | " | 50 |
| Assafœtida, fair | " | 45 |
| Select | " | 70 |
| Benzoin | " | 1 00 |
| Catechu, true | " | 16 |
| Gamboge | " | 1 50 |
| Powdered | " | 1 60 |
| Guiac | " | 55 |
| Powdered | " | 65 |
| Euphorbium | " | 30 |
| do powdered | " | 45 |
| Kino | " | 90 |
| Mastic | " | 4 50 |
| Myrrh, Turkey | " | 60 |
| do powdered | " | 70 |
| Sanderach | " | 65 |
| Shellac, Campbell's | " | 55 |
| Good Native Orange | " | 45 |
| Bleached | " | 85 |
| Tragacanth, White Flake, No. 1 | " | 1 75 |
| No. 2 | " | 1 50 |
| Sorts | " | 45 |
| Hemp Seed, by bbl | " | 8 |
| Less than bbl | " | 7 |
| Hoffman's Anodyne, P. & W. | " | 60 |
| Insect Powder | " | 1 25 |
| Iodine Resublimed, 1 lb. bottles | " | 6 75 |
| 1 oz. vials | per oz. | 48 |
| Ipecac, powdered, pure | per lb. | 3 75 |
| Iron, Carb. Precip., P. & W. | " | 25 |
| Hydrogen; P & W | " | 2 75 |
| Iodide Syrup | " | 70 |
| Muriated Tincture | " | 44 |
| Pyrophosphate | " | 1 50 |
| Isinglass, Amer., (Norwood & Manning's) | " | 1 25 |
| Per barrel | " | 1 10 |
| Jalap Root, powdered | " | 2 20 |
| Juniper Berries, by bag | " | 5 |
| Less thrn bag | " | 7 |
| Kreosote | " | 1 25 |
| Lemon Peel | " | 25 |

| | | |
|---|---|---|
| Laurel Leaves | per lb. | 12½ |
| Liquorice Extract, Calabria, gen., P. & S | " | 45 |
| Liquorice Extract, Calabria, gen., Barraco | " | 45 |
| Root, select | " | 16 |
| Powdered | " | 20 |
| Sicily | " | 24 |
| Lobelia Seed | " | 60 |
| Lunar Caustic, Cones Squibb's. | per oz. | 2 20 |
| Lupuline | per lb. | 1 00 |
| Lycopodium | " | 85 |
| Mace | " | 1 65 |
| Magnesia Carb. 2 oz. papers, Pattison's | " | 38 |
| 4 oz. papers, Pattison's | " | 37 |
| 2 do Jenning's | " | 48 |
| 4 do do | " | 44 |
| Calcined | " | 75 |
| do Jenning's 1 lb. bottles | " | 1 45 |
| Manganese, black, Oxide | " | 8 |
| Manna, sorts | " | 75 |
| Large Flake | " | 2 40 |
| Small Flake | " | 1 75 |
| Marble Dust | per bbl. | 3 50 |
| Mercurial Ointment, P. & W., ½ mercury | per lb. | 70 |
| P. & W., ⅓ mercury | " | 55 |
| Morphine, Sulph., P. & W., vials included | per oz. | 9 25 |
| Moss, Iceland | per lb. | 15 |
| Irish, select, by bbl | " | 10 |
| do No. 1, by bbl | " | 7 |
| Musk, Chinese, oz. cans | per oz. | 1 00 |
| Tonquin, true, in grain | " | 38 00 |
| do do in pod | " | 16 00 |
| Mustard, Ground Coleman's, g. | per lb. | 55 |
| In cans, imitation | " | 35 |
| Taylor's in cans | " | 75 |
| do in foil, S. F. | " | 80 |
| do in foil, D. S. F. | " | 85 |
| Seed, white, English | " | 19 |
| do American | " | 14 |
| Seed, black, English | " | 18 |
| do American | " | 12 |
| W ld's, 4 and 6 lb. cans | " | 45 |
| Nitrate of Silver, pure | per oz. | 1 33 |
| Nutgalls, Aleppo | per lb. | 44 |
| do do powdered | " | 45 |
| Nutmegs, No. 1 | per lb. | 1 45 |
| Nux Vomica | " | 20 |
| do powdered | " | 38 |
| Oil, Almond, sweet | " | 95 |
| do bitter, essential | per oz. | 1 65 |
| Amber, crude | per lb. | 60 |
| do rectified | " | 1 40 |
| Anise | " | 4 50 |
| Bergamot, Sanderson's | " | 7 50 |
| Cassia | " | 4 00 |
| Caraway | " | 3 00 |
| Citronelle | " | 2 75 |
| Cajuput | " | 2 25 |
| Citronelle, native | " | 2 75 |
| do Winter's | " | 3 25 |
| Cloves | " | 3 20 |
| Cod Liver, Hegeman & Co's | per doz. | 7 50 |
| Cod Liver, Rushton's | " | 8 00 |
| Cod Liver, bulk, pure | per gal. | 2 35 |
| Copaiba | per lb. | 4 25 |
| Coriander | per oz. | 2 50 |
| Croton, English | per lb. | 3 00 |
| Cubebs, P. and W. | " | 4 50 |
| Cumin | per oz. | 65 |
| Ergot | " | 30 |
| Erigeron | per lb. | 4 00 |
| Fennel | per lb. | 3 00 |
| Hemlock | per lb. | 50 |
| Juniper | " | 1 40 |
| Juniper Berries | per oz. | 50 |
| Lemon, Opt, Sanderson's | per lb. | 4 75 |
| do Grass Winters | per oz. | 55 |
| Lavender, Garden. fine | per lb. | 1 75 |
| Lavender, Opt. Chiris | " | 4 25 |
| Mace, expressed | " | 3 00 |
| Mustard, essential | per oz. | 2 25 |
| Myrrbane | per lb. | 2 75 |
| Neroli, Chiris | per oz. | 4 75 |
| Nutmegs | " | 75 |
| Olive, Marseilles, quarts | per doz. | 6 75 |
| do do pints | " | 4 00 |
| do Daccosini, quarts | " | 6 00 |
| do do pints | " | 3 25 |
| do Bordeaux, quarts | " | 14 50 |
| do do pints | " | 8 00 |
| Orange Bitter | per lb. | 7 00 |
| Orange Flowers | " | 5 50 |
| Origanum, pure | " | 1 40 |
| do commercial | " | 65 |
| Pachouly | per oz. | 5 00 |
| Pennyroyal | per lb. | 3 50 |
| Peppermint, pure | " | 6 26 |
| Rhodium | per oz. | 85 |
| Rosemary | " | 1 35 |
| Rose Geranium, Chiris | " | 1 25 |
| do Kisanlink, pure | " | 10 25 |
| do commercial | " | 5 00 |
| Sassafras | per lb. | 1 15 |
| Savin | " | 1 75 |
| Spearmint | " | 6 00 |
| Tansy | " | 5 50 |
| Wintergreen, pure | " | 4 75 |
| Wormseed | " | 4 00 |
| Wormwood | " | 7 00 |
| Opium, Turkey | " | 12 15 |
| Purkey, powdered | " | 17 00 |
| Orange Peel | " | 14 |
| Ground | " | 18 |
| Orris Root, select | " | 20 |
| Powdered | " | 25 |
| Peruvian Bark, Red Quill, P. and W. | " | 1 75 |
| Red Quill, powdered, P. & W. | " | 2 05 |
| Peruvian Bark, Rubingosa, powdered | " | 1 00 |
| Yellow Quill | " | 30 |
| do do powdered | " | 40 |
| Pink Root | " | 35 |
| Piperine | per oz. | 1 65 |
| Potassa Bromide | per lb. | 1 50 |
| Acetate | " | 85 |
| Carb. (Sal. Tartar) | " | 20 |
| Potash, crude, in casks, No. 1 | " | 7¾ |
| Potash, Chlorate, French | " | 75 |
| do English | " | 55 |
| Prussiate | " | 44 |
| Bichromate | " | 24 |
| Potassium Iodide, Pfizer's | " | 5 25 |
| A. and B. | " | 5 60 |
| Cyanide, fused | " | 80 |
| Plaster Paris, ordinary | per bbl. | 4 50 |
| do do Dentists' | " | 5 00 |
| Quinine, Sulph., P. and W. | per oz. | 2 40 |
| Sulph, French | " | 2 40 |
| Quicksilver | per lb. | 90 |
| Red Precipitate | " | 1 18 |
| Rhubarb Root, E. I., select | " | 3 00 |
| Powdered, select | " | 3 20 |
| English | " | 2 65 |
| English, powdered | " | 2 75 |
| Rice Flour, 1 lb. packa6es | " | 15 |
| Rochelle Salts, P. and W. | " | 50 |

| | | |
|---|---|---|
| Rose Leaves, damask | per lb. | 2 75 |
| Rosin, pale | " | 4 @ 5 |
| Sago, Pearl, by case | " | 14 |
| Less than case | " | 15 |
| Sarsaparilla, Honduras | " | 58 |
| Honduras, ground | " | 63 |
| Sassafras bark, by bbl | " | 12½ |
| Less than bbl | " | 14 |
| Saffron, American | " | 2 75 |
| Spanish | " | 17 50 |
| do | per oz. | 1 20 |
| Salacin | per oz. | 55 |
| Saltpetre, crude | per lb. | 14 |
| Hudson | " | 12½ |
| Pure Crystals (by 100 lb. keg) | " | 17 |
| Pure Crystals (less than keg) | " | 19 |
| Powdered | " | 28 |
| Santonine, P. and W. | per oz. | 28 |
| Seidlitz Mixture, P. and W. | per lb. | 44 |
| Seneka Root | " | 65 |
| Senna, Alexandria, sifted | " | 55 |
| do ordinary | " | 45 |
| E. I. | " | 30 |
| Tinnevelly | " | 38 |
| Snuff, Scotch | " | 70 |
| Macaboy, rose scented | " | 75 |
| Soap, Castile, Mottl'd, genuine, by box | " | 17 |
| Castile, white, by box | " | 26 |
| Soda, Bicarb., Newcastle, by keg | " | 6½ |
| Less than keg | " | 8 |
| Sal, (by bbl., 500 lbs) | " | 3¼ |
| Less than bbl | " | 4 |
| Soda Ash, best brands, in c'ks | " | 4 |
| Spermaceti | " | 58 |
| Squills, Root | " | 15 |
| Strychnia, Crystals, vials included, P. and W. | per oz. | 3 35 |
| Powdered | " | 3 10 |
| Sugar Lead, white | per lb. | 37 |
| Milk, powdered | " | 65 |
| Sulphur, Flour, by bbl | " | 6 |
| Less than bbl | " | 7 |
| Sweet Spirits Nitre, 4 F | per lb. | 42 |
| do do 3 F | " | 33 |
| Sweet Quinine | per oz. | 2 00 |
| Tamarinds | per lb. | 12 |
| Tannin, bottle included | per oz. | 35 |
| Tapioca, Rio, bg bag | per lb. | 13 |
| Less fhan bag | " | 16 |
| Tar, Carolina, pure | per bbl. | 6 50 |
| Tincture Presses, ½ gall | each | 10 50 |
| do 1 do | " | 15 00 |
| Tonqua Bean | per lb. | 80 |
| Augostura | " | 1 15 |
| Uva Ursi, French | " | 15 |
| Valerian Root, English | " | 75 |
| do German | " | 33 |
| Vanilla Beans, short | " | 12 50 |
| do long | " | 14 50 |
| Venice Turpentine | " | 28 |
| White Wax, pure | " | 90 |
| Zinc, Sulphate | " | 12 |

### White Leads, French and American Zincs, Tieman's Colors, Varnishes, Etc.

| | | |
|---|---|---|
| Alcohol, 98 per cent., by bbl | per gal. | $2 40 |
| Axle Grease, National | per doz. | 1 50 |
| Frazer's, in wood boxes | " | 1 50 |
| do in tin cans, small | " | 2 50 |
| do do med. | " | 4 25 |
| do do large | " | 6 75 |
| Bidwells | " | 1 50 |
| Benzole, deodorized, by bbl. | per gal. | 16 @ 30 |
| Black Drop, dry, American | per lb. | 15 |
| Dry, English | " | 15 |
| In oil, 1 lb. cans, English | " | 20 |
| Paint, Tieman's, in oil, 1 lb. cans | " | 12½ |
| Blue, Ultramarine (H. B b'nd) | " | 35 |
| Ultramarine, in oil | " | 35 |
| Pruss., Tieman's, Paris, dry | " | 80 |
| do Paris, in oil | " | 70 |
| do Chinese, dry | " | 85 |
| do A, dry | " | 70 |
| do A, in oil | " | 55 |
| do Manh'n, dry | " | 65 |
| do Manh'n, in oil | " | 50 |
| do Knick'r, dry | " | 52 |
| do do in oil | " | 40 |
| Brown, Spanish, by bbl | " | 2 |
| Less than bbl | " | 3 |
| Gold leaf, XX, deep | per pack | 9 75 |
| do pale | " | 9 00 |
| Green, chrome, Tieman's, pure dry, No 1 | per lb. | 18 |
| Tieman's, pure, in oil, No. 1 | " | 19 |
| do Manhattan, dry | " | 16 |
| do Manhatt'n, in oil | " | 17 |
| do Knickerb'r, dry | " | 14 |
| do do in oil | " | 15 |
| Green, Paris, Tieman's, dry | " | 30 @ 40 |
| do do in oil | " | 30 @ 50 |
| do do ex. permanent, in oil | " | 40 |
| Hampden, dry | " | 24 |
| Hampden, in oil | " | 25 |
| Magnesia, Heineman & Steiner's, dry | " | 25 |
| H. & S.'s in oil | " | 26 |
| Marseilles, Wood's, in oil | " | 28 |
| Lamp Black, Eddy's, refined, by bbl | " | 30 |
| Less than bbl | " | 32 |
| 1 oz. papers, per 100 | | 1 00 |
| Common, assorted | " | 9 |
| ¼ lb. papers | " | 11 |
| ½ lb. papers | " | 12 |
| Germantown, assorted | " | 15 |
| do ¼'s | " | 18 |
| do ½'s | " | 16 |
| do 1's | " | 13 |
| Lead, White, Jewett's | per 100 lbs. | 13 75 |
| Atlantic | " | 13 75 |
| B. L. Fahnestock's | " | 13 75 |
| Fahnestock, Hazlett & Schwart's | " | 13 75 |
| Hall, Bradley & Co.'s | " | 13 75 |
| Collier & Co.'s, pure | " | 13 00 |
| Collier & Co.'s, str'tly pure | " | 13 75 |
| Garden City | " | 11 50 |
| Diamond | " | 10 50 |
| Lead, Sterling | " | 8 00 |
| Continental | " | 11 50 |
| Assorted cans, 1 to 5 lbs. | " | 12 00 |
| Dry, white | " | 13 50 |
| Red, American | " | 12 50 |
| English, 112 lb. kegs | " | 13 00 |
| Mineral Paint, Ohio, by bbl | per lb. | 2¾ |
| Less than bbl | " | 3½ |
| Winter's, by bbl | " | 3¼ |
| Less than bbl | " | 4 |
| Ochre, Oxford, Stone, in oil | " | 17 |
| Oil, Carbon, Alex. Schofield & Co., by bbl | per gall. | 33 |
| Castor, E. I., by case | " | 3 15 |
| do American, by bbl | " | 3 00 |
| Lard, extra, by bbl | " | 1 45 |
| do No. 1, do | " | 1 30 |
| do No. 2, do | " | 1 12 |

Linseed raw, by bbl.........per gal. 1 03
Linseed, boiled, by bbl..... " 1 08
Machinery, Eclipse, by bbl. " 45
do West Va. do " 60
Neatsfoot, by bbl............ " 1 20
Olive, bulk, pure Malaga... " 1 85
do do do Marseilles " 4 00
do Union.................. " 1 45
Sperm, winter bleached, by bbl........................ " 2 40
Tanners' Bank, by bbl...... " 1 06
Whale, winter bleach'd, by bbl.......................... " 1 25
Putty, bulk, 100 lb tubs.........per lb. 4½
In bladders, by bbl......... " 4⅞
Less than bbl.............. " 5½
Red Indian, dry..................... " 16
In oil.............................. " 22
Rose Pink, Tieman,s............... " 18
Sand Paper, Baeder & Adamson's, Flint...............per ream 5 50
National, Flint, assorted... " 4 50
Metropolitan, Flint, ass'd.. " 3 75
Sienna, raw, dry....................per lb. 7
Tieman's, Manhat'n, in oil " 18
Tieman's, Knickerbocker, in oil.......................... " 16
Burnt, dry...................... " 7
Burnt, Tieman's, Manhattan, in oil................ " 18
Burnt, Tieman's, Knickerbocker, in oil .............. " 16
Turpentine, Spirits, by bbl......per gal. 50
Less than bbl................. " 58
Umber, raw, dry....................per lb. 7
Tieman's, Manhattan, in oil " 17
do Knickerb'r, in oil " 15
Burnt, dry...................... " 7
Burnt, Tieman's Mahattan, in oil............................ " 17
Burnt, Tieman's, Knickerbocker, in oil .............. " 15
Vandyke Brown, dry............. " 12
Tieman's, in oil .............. " 22
Varnish, coach, No. 1, Turpentine (Smith's)...............per gal. 3 00
Coach, ex., Turp., Tilden's " 4 00
No. 1, do do ... " 2 75
Furn., No. 1, do do ... " 2 25
Furn., No. 2, do do ... " 2 10
Damar, Turpentine.......... " 2 65
Damar, Benzole............... " 2 40
Japan, Benzole................ " 90
Japan, Turpentine ........... " 1 20
Venetian Red, Tieman's, in oil..per lb. 12½
English, Cookson's, by bbl. " 3¾
English, less than bbl...... " 4
American, by bbl............ " 2½
Less than bbl.............. " 3½
Vermillion, Amer'n, Tieman's, dry......................... " 27
American, Tieman's, in oil " 29
do Hamden.......... " 27
Tieman's California, pale... " 1 25
do do deep... " 1 20
Chinese ......................... " 1 30
Trieste ........................... " 1 25
English A. & B., pale........ " 1 25
English A. & B., deep ...... " 1 20
Whiting, Spanish, by bbl........ " 2¾
Less than bbl.................. " 3½
White, Paris, by bbl................ " 4
Less than bbl................ " 5
Yellow Chrome, dry, Tieman's pure ......................... " 28
Tieman's Manhattan, dry. " 20
do Knickerb'r, dry.. " 14

Tieman's Pure, in oil ........per lb. 29

do Manhatt'n, in oil " 21

do Knickerb., in oil " 15

Yellow Ochre, Am., by cask.... " 2¼

Less than cask................ " 3

Rochelle, by cask............ " 3

Less than cask............. " 3½

Havre, by cask ............. " 2¾

Less than cask............. " 3¼

French, in oil, ass'd cans... " 12½

Zinc, Veille. Montague, Red Seal, in oil.................... " 16⅛

Veille, Montague, R. S., in oil ....................................... " 17½

Zinc, white, in oil, New Jersey " 10 50

do do Penn. and Lehigh ......................... " 11 50

### Window Glass.

WINDOW GLASS—all sizes—in good shipping order.

Discouut, 50 per cent.

| Size. | 1st Qual. | 2d Qual. |
|---|---|---|
| 6x 8 to 8x10 | $ 6 50 | $5 50 |
| 8x11 to 10x15 | 7 00 | 6 00 |
| 8x16 to 12x18 | 8 00 | 7 00 |
| 10x20 to 16x24 | 8 50 | ...... |
| 10x26 to 20x28 | 10 50 | ...... |
| 20x30 to 24x30 | 12 00 | ...... |
| 24x31 to 24x36 | 13 00 | ...... |
| 25x36 to 30x44 | 15 00 | ...... |
| 30x46 to 32x46 | 16 00 | ...... |

Double thick, double price.

### Dye Stuffs. Dye Woods and Chemicals for Dyers and Woolen Manufacturers.

Acid, Picric, Cryst.................per lb. $ 3 50
Pyroligneous..................per gal. 25
Aniline, black ........................per lb. 1 25
do .........................per oz. 20
Blue, Parme.......... .. .......per lb. 9 00
do do ....................per oz. 75
do Violet.....................per lb. 10 50
do do .....................per oz. 1 00
do Hoffman's ......... .....per lb. 15 00
do do ...............per oz. 1 25
Brown ....... ....................per lb. 9 00
do ..........................per oz. 75
Green, for wool...............per lb. 30 00
do do ...............per oz. 2 50
do for silk..................per lb. 45 00
do do ..................per oz. 3 00
Orange..........................per lb. 9 00
do .........................per oz. 75
Purple ..........................per lb. 13 50
do .........................per oz. 1 20
do Royal ..................per lb. 15 00
do do ..................per oz. 1 25
Red ..............................per lb. 8 00
do ..............................per oz. 60
Scarlet .........................per lb. 8 00
do ..........................per ox. 75
Violet.............................per lb. 10 50
do ...............................per oz. 1 00
Yellow ..........................per lb. 12 00
do ..........................per oz. 1 00
Aqua Fortis, carboy...............per lb. 16
do less.................. .. " 20
Barwood...................by bbl. per lb. 4½
Brazil Wood ............. " " 15
Camwood, Commerc'l.. " " 8
do pure.......... " " 12
Ebony Wood............. " " 6
Fustic, Cuba............. " " 3½
Hypernic .................. " " 7½

| | | | |
|---|---|---|---|
| Lima Wood | by bbl. | per lb. | 8 |
| Logwood, 'Campeachy' | " | " | 3 |
| Nicwood | " | " | 4 |
| Peachwood | " | " | 6 |
| Quercitron | " | " | 3½ |
| Red Sanders | " | " | 5½ |
| Sapan Wood | " | " | 10 |
| Tumeric | " | " | 15 |

### Sundries.

| | | |
|---|---|---|
| Alum, by bbl | per lb. | $ 4½ |
| Annatto, best Para | " | 1 65 |
| do Eng. Roll | " | 85 |
| Argols, refined, powdered | " | 26 |
| Black Lead, E. I., powdered | " | 15 |
| German, powdered | " | 12 |
| Bleaching powder, by cask | " | 5 |
| Candles, Parrafine, 4's and 6's | " | 36 |
| Sperm, 4's and 6's | " | 56 |
| Patent Wax, 4's and 6's | " | 68 |
| Carmine, No. 40 | per oz. | 1 30 |
| China Clay | per lb. | 3½ |
| Cutch, " Pegue | " | 16 |
| Flavine | " | 1 00 |
| Fuller's Earth | " | 5 |
| Gambia, or Japonica, bales | " | 7½ |
| Indigo, Bengal, 1st quality | " | 8 50 |
| do 2nd do | " | 3 00 |
| Indigo, Gautemala | " | 2 30 |
| Macras. 1st quality | " | 1 65 |
| do 2nd do | " | 1 55 |
| Manilla 1st do | " | 1 65 |
| do 2nd do | " | 1 55 |
| Extract | " | 65 |
| Carmine | " | 5 50 |
| Irish Moss, by bbl | " | 7 |
| Iron Liquor, by gal | " | 45 |
| Lac Lye | " | 60 |
| Litharge | " | 14 |
| Logwood, Extract, 12 lb. boxes | " | 17 |
| Madder, Best Dutch, casks | " | 21 |
| do do by bbl | " | 22 |
| Notgalls, Aleppo | " | 44 |
| Orchill Paste | " | 38 |
| Pearl Ash, by bbl | " | 15 |
| Sal Acetosella | " | 65 |
| Ammoniæ | " | 20 |
| Tartrr | " | 20 |
| Sumac, Sicily | " | 8 |
| Lead Seal | " | 10 |
| Tartar, Cream of, pure, cryst | " | 48 |
| do do do powd | " | 50 |
| do Red, or Argols | " | 26 |
| Tin Bar | " | 49 |
| Muriate, cryst | " | 48 |
| do solution | " | 24 |
| Oxymuriate do | " | 30 |
| Per-chloride | " | 75 |

### Superfine (XX) Straight or Taper Corks.

| | |
|---|---|
| No. 0 | 20 |
| 1 | 24 |
| 2 | 28 |
| 3 | 32 |
| 4 | 37 |
| 5 | 42 |
| 6 | 52 |
| 7 | 85 |
| 8 | 90 |
| 9 | 1 15 |
| 10 | 1 30 |
| 11 | 1 40 |
| 12 | 1 60 |
| 13 | 1 75 |
| 14 | 1 90 |
| 15 | 2 20 |
| 16 | 2 45 |
| 17 | 2 75 |
| 18 | 2 90 |

### Fine (X) Straight or Taper Corks.

| | |
|---|---|
| No. 0 | 10 |
| 1 | 12 |
| 2 | 15 |
| 3 | 18 |
| 4 | 20 |
| 5 | 25 |
| 6 | 30 |
| 7 | 38 |
| 8 | 50 |
| 9 | 60 |
| 10 | 70 |
| 11 | 80 |
| 12 | 90 |
| 13 | 1 05 |
| 14 | 1 25 |
| 15 | 1 35 |
| 16 | 1 50 |
| 17 | 1 65 |
| 18 | 1 75 |

### Specie or Flat Corks.

| | |
|---|---|
| 1 inch | 40 |
| 1⅛ " | 45 |
| 1¼ " | 50 |
| 1⅜ " | 60 |
| 1½ " | 65 |
| 1⅝ " | 80 |
| 1¾ " | 1 00 |
| 1⅞ " | 1 20 |
| 2 " | 1 35 |
| 2⅛ " | 1 40 |
| 2¼ " | 1 55 |
| 2⅜ " | 1 70 |
| 2½ " | 1 85 |
| 2⅝ " | 2 10 |
| 2¾ " | 2 35 |
| 2⅞ " | 2 60 |
| 3 " | 2 85 |
| 3¼ " | 3 20 |
| 3½ " | 3 50 |
| 3¾ " | 4 00 |
| 4 " | 4 50 |

### Short Taper (XX) Corks.

| | |
|---|---|
| No. 2 | 20 |
| 3 | 25 |
| 4 | 30 |
| 5 | 35 |
| 6 | 45 |
| 7 | 60 |
| 8 | 70 |
| 9 | 90 |
| 10 | 1 00 |
| 11 | 1 15 |

